# Identification of coral endosymbionts of Veedhalai and Mandapam coasts of Palk Bay, India using small subunit rDNA

**DOI:** 10.6026/97320630018318

**Published:** 2022-04-30

**Authors:** Rajesh Kannan Murugesan, Ramkumar Balakrishnan, Sivakumar Natesan, Sridhar Jayavel, Ramakritinan Chockalingam Muthiah

**Affiliations:** 1 Department of Marine and Coastal Studies, School of Energy, Environment and Natural Resources, Madurai Kamaraj University, Madurai - 625021, Tamil Nadu, India; 2 Department of Molecular Microbiology, School of Biotechnology, Madurai Kamaraj University, Madurai - 625021, Tamil Nadu, India; 3 Department of Biotechnology, Department of Distance Education, Madurai Kamaraj University, Madurai - 625021, Tamil Nadu, India

**Keywords:** Scleractinian Corals, Endosymbionts, 18S nrDNA, Durusdinium, Cladocopium, Thermotolerant, Palk Bay

## Abstract

Coral endosymbionts act as a bio-indicator of coral ecosystem under extreme environmental conditions. The health of the coral ecosystem depends on the endosymbiont cell density of the coral hosts. Therefore, it is of interest to analyze ten coral fragments found to be under the genera Acropora, Favites, Favia, and Porites collected at various locations from Veedhalai to Mandapam, southeast coast of India during January 2019 to March 2019. The zooxanthellae cell count ranged between 4.08 (Porites sp.9) and 13.75x105 cells cm2 -1 (Favites sp.3). This indicates the health of the corals in the region. The genus (clade) level identification of endosymbionts was detected using the host excluding primers of small subunit DNA (nssrDNA). Bidirectional sequencing of 18S nrDNA gene (SSU) of all ten coral fragments show that the Veedhalai corals is associated with the genus Durusdinium (Clade D) but the corals of Mandapam is associated with the genera, Cladocopium (Clade C) and Durusdinium (Clade D). It is known that the thermal stress has negative impact on coral reef ecosystem of the world. The dominance of the genus Durusdinium in the scleractinian corals of Palk Bay may be due to frequent exposure to thermal stress. This thermotolerant endosymbionts is opportunistic. Thus, the corals of Veedhalai and Mandapam coasts, Palk Bay, India are necessarily packed with thermotolerant endosymbionts enabling conservation.

## Background:

Zooxanthellae, the unicellular dinoflagellates of the familySymbiodiniacea, are free living or have a special symbiotic relationship existing with foraminifera, scleractinian corals, octocorals, sea anemones, molluscs, Platyhelminthes and porifera for the past 250 million years [1-4]. Zooxanthellae are a vital nutrient cycling within the host corals [5] and a prolonged loss can lead to coral mortality [6-8]. Most cnidarians preferentially establish and maintain a stable symbiosis with either a specific Clade of endosymbionts [9] or a subset of the Clades that vary with environmental gradients such as light intensity, temperature etc. [10-12]. The endosymbionts of the family Symbiodiniacea was formerly classified into nine genetic Clades such as A-I [1,13] which were re-classified from a single genus Symbiodinium as being equivalent to the genera such as Symbiodinium (A), Breviolum (B), Cladocopium (C), Durusdinium (D), Fugacium (E) and Gerakladium (F) [14]. The endosymbionts are extremely diverse which have many evolutionarily divergent lineages [15] and they comprise of many unrecognized species [16]. Earlier studies revealed all endosymbionts grouped under a single species, Symbiodinium microadriaticum (Freduenthal) [17]. Later the biochemical, morphological, karyotyping motility patterns and DNA/DNA hybridisation studies proved endosymbionts belong to different species and strains [18-19]. This was confirmed by the application of various molecular tools (sequencing of nssrDNA, nlsrDNA, ITS rDNA, and clsrDNA [20, 24-check with author]. Phylogenetic clades are classified based on rDNA and chloroplast DNA [25]. Ribosomal RNA is universal and composed of highly conserved as well as variable domains [26-27]. Therefore, it is of interest to analyze ten coral fragments found to be under the genera Acropora, Favites, Favia, and Porites collected at various locations from Veedhalai toMandapam, southeast coast of India during January 2019 to March 2019.

## Materials and Methods:

### Coral sampling:

The Palk Bay represents the region between southeast coast of India and northwest coast of Sri Lanka, and separated by Pamban Pass of Gulf of Mannar in south and extends up to Kodiakarai coast in the north. In this study, totally ten coral fragments were sampled in the Palk Bay; of these, two fragments from Veedhalai (9.298495°N, 79.1014012°E) and another eight from Mandapam (9.2959824°N, 79.1291909°E) (Figure 1 - see PDF) between January 2019 and March 2019. The fragments were sampled using SCUBA diving techniques using a hammer and chisel. The locations were marked using Garmin GPSMAP 78sc. During sampling, the underwater photographs of corals were taken using Nikon DSLR camera (Model # D 7000) with Ikelite underwater housing (#6801.7). The in-situ coral photographs were used for identification up to species level based on the morphological characteristics [28-31 - check with author] and they were preserved in the Marine Field Research Station of Madurai Kamaraj University located at Pudumadam in Ramnad District, Tamil Nadu, India.

### Isolation of Symbiont from corals:

For symbiont cell density analysis, symbionts were isolated slightly modified method as described by Rowan and Power [32 - check with author] and Chen et al. [24 - check with author]. Coral fragment was individually homogenized in Zooxanthellae Isolation Buffer and filtered through 125µm mesh to remove large pieces of animal tissues before recovering zooxanthellae by centrifugation at 10000g for 1 min. The yellowish-brown pellet was frequently washed, separately placed in a vial and stored at -20°C for cell density analysis and molecular identification.

### Symbiont cell density analysis:

The symbiont cell density was analyzed using the method described by Lasker [[33].The symbiont pellet was diluted with 1 mL of PBS and tapped gently or vortexed for uniform mixing. About 40 µL of suspension was placed in Neubauer Improved Tiefe Depth Profoundeur (0.100 mm) haemocytometer, and viewed under 40X magnification with Labomed 400X Trinocular light microscope. Symbiont cell density was calculated as given below:

Cell count = (No. of the cells counted in the central squares x dilution factor)/Area of the small square (cm^2^)

### Extraction of symbiont DNA, PCR amplification and RFLP analysis:

The stored symbiont pellet was treated with DNA isolation buffer and incubated in a water bath at 65°C for 60 minutes. Proteinase K (0.5mg mL-1) was added and kept for overnight incubation at 37°C in a water bath. Lysates were extracted once with chloroform: isoamyl alcohol (24:1) and centrifuged twice at the speed of 12,000 rpm using REMI cooling centrifuge. The aqueous phase was collected and an equal volume of 4M lithium chloride and two volumes of ice-cold isopropanol were added. Again, the samples were incubated at -80°C for 2 hours and then centrifugation was carried out at 12,000 rpm for 10 minutes. The supernatant was discarded and the pellets were washed with 70% ethanol. Finally, the supernatant was discarded and the pellet containing symbiont DNA was air-dried. It was dissolved using sterile Milli-Q water and stored at -20°C in a refrigerator. After the isolation of DNA in corals, the integrity of DNA was checked by 0.8% Agarose Gel Electrophoresis and the quantity of DNA was also checked by using Thermo Scientific ND-2000 Nano drop. An approximate 1600-bp fragment of SSU was amplified with host excluding primers described by Rowan and Power [32 - check with author] (Ss 5 forward-5’-GGTTGATCCTGCCAGTAGTCATATGCTTG-3' and Ss 3 Z reverse-5'-AGCACTGCGTCAGTCCGAATAATTCACCGG-3). The PCR amplification conditions for SSU consisted initial denaturation at 94°C for 3 minutes, denaturation at 94°C for 1 minute, annealing at 56°C for 1 minute, strand elongation at 72°C for 2.30 minutes and final extension at 72°C for 8 minutes. For RFLP analysis, the PCR products of SSU were digested with restriction enzyme of Taq I. After digestion, the products were run in 2% agarose gel electrophoresis to visualize the digested fragments.

### Sequencing and Phylogenetic analysis:

The bidirectional sequencing of PCR products of 18S rDNA was done from Macrogen Inc, Korea. The PCR products were cleaned using a PCR clean-up kit ExoSAP-IT and sequenced. Sequencing was done using the Dye Terminator technique with ABI PRISM 3730XL Analyzer. Nucleotide database was searched with the sequences obtained with NCBI BLAST (Blastn) tool (http://www.ncbi.nlm.nih.gov/BLAST) [34] and the GenBank Accession numbers were obtained. The phylogenetic relationship was performed using MEGA-7 Software [35]. Genetic distances among each isolate and out-group were calculated based on the Maximum Likelihood Nearest Neighbor Interchange method hence this method is used for phylogenetic tree reconstruction.

## Result & Discussion:

### Symbiont Cell density:

The health of the coral reef ecosystem depends mainly on the symbiont density as well as its association with coral host. It is a potential diagnostic indicator of reef corals under stress [36]. The drastic difference in cell density leads to instability in symbiosis with coral host and the stability means that the zooxanthellae density in coral hosts relatively constant under a given set of environmental conditions and the symbiotic partners do not change [37]. For assessing the health of reef corals, the coral symbiont cell density of Veedhalai (two fragments) and Mandapam (eight fragments) of Palk Bay coast estimated was given in Figure 2(see PDF). The present study showed that there was a great variation in symbiont cell density between corals as well as the chosen sites. The present study results were in agreement with the findings of Fitt et al. [38] They reported the symbiont cell density ranged between 1 x 106 cm-2 -1 to 2 x 106 cells cm-2 -1 of coral surface and these variations may be due to the temporal and spatial scales. Fagoonee et al. [39] and Fitt et al. [38] suggested that the cell density of tropical corals found to be higher during low light months (winter season).

Data shows that the highest and lowest cell densities were reported from the corals sampled at Mandapam coast. Even though, there were variations in symbiont cell density of corals such as Porites sp. 9, Favites sp. 2 and Favia sp.4 sampled from Mandapam region i.e., 4.08 (±1.18), 5.58 (±1.13)and 5.33 (±0.52) x 105 cells cm2 -1 respectively, it shows insignificant difference. The Favites sp. 4 sampled at Mandapam were at 13.75 (±0.31) x 105 cells cm2 -1. Although Acropora sp. 10 and Acropora sp. 11 collected from Veedhalai were in the range of 8.17 (±1.01) and 9.67 (±1.63) x 105 cells cm2 -1 respectively.

The present study concludes that symbiont cell density of corals of Palk Bay was much higher as reported by Oladi et al. [36]. As the cell density of 2.6 x 105 cells cm2 -1 is considered to be a healthy reef. The present study shown a significant difference of chosen the sampling sites indicating the reef corals of Veedhalai and Mandapam of Palk Bay found to be healthy. Hence, more studies are needed to analyse the variability of symbiont cell density to study the Coral bleaching under environmental stress [37].

### Symbiont genetic diversity:

The studies on the molecular diversity of coral associated symbiont of Indian corals are limited [40]. Hence, the present study was mainly investigated to bring out the genus (Clade) level identification of the endosymbionts to the family Symbiodiniaceae harboured with the scleractinian corals of Mandapam and Veedhalai coasts, Palk Bay using SSU rDNA gene sequencing. The PCR amplification of genomic DNA of SSU of symbionts of Veedhalai and Mandapam corals produced a single amplicon of ∼1660 bp which were amplified without host cells (Figure 3 - see PDF). The restriction digestion was differentiated symbiont (zooxanthella) from animal DNA. The symbiont 18S rDNA region has a restriction site at TCGA. The restriction digestion of these amplicons using Taq I yielded two major RFLP profiles revealed two fragments of 890 bp and 710 bp or other two fragments of 890 bp and 500 bp or-else a mixed RFLP patterns being observed [32 -check with author]. On contrary, a single RFLP pattern with two fragments (710 bp and 250 bp) was reported by Chen et al. [24 - check with author] In the present report a single RFLP pattern with 800/250 bp of the endosymbionts from Mandapam and Veedhalai regions, may be a genotype mixture of Clade E and Clade D symbionts (Figure 4 - see PDF). The sequence data analyzing BLAST-N results disclosed >99% similarity with genus Durusdiniumandgenus Cladocopium collected from the corals at respective region. The NCBI GenBank accession numbers obtained for the submitted sequences were MN874265 - MN874274 (Table 1 - see PDF).

Veedhalai and Mandapam corals produced a single amplicon of ∼1660 bp which were amplified without host cells (Figure 3 - see PDF). The restriction digestion was differentiated symbiont (zooxanthella) from animal DNA. The symbiont 18S rDNA region has a restriction site at TCGA. The restriction digestion of these amplicons using Taq I yielded two major RFLP profiles revealed two fragments of 890 bp and 710 bp or other two fragments of 890 bp and 500 bp or-else a mixed RFLP patterns being observed [32 - check with author]. On contrary, a single RFLP pattern with two fragments (710 bp and 250 bp) was reported by Chen et al. [24 - check with author] In the present report a single RFLP pattern with 800/250 bp of the endosymbionts from Mandapam and Veedhalai regions, may be a genotype mixture of Clade E and Clade D symbionts (Figure 4 - see PDF). The sequence data analyzing BLAST-N results disclosed >99% similarity with genus Durusdiniumandgenus Cladocopium collected from the corals at respective region. The NCBI GenBank accession numbers obtained for the submitted sequences were MN874265 - MN874274 (Table 1 - see PDF).

### Genetic Diversity:

The nssrDNA sequencing studies on endosymbionts isolated from the corals of Palk Bay indicate the presence of genus Cladocopium (Clade C) and genus Durusdinium (Clade D) in the chosen region particularly in Mandapam region, both species found wherein only the genus Durusdinium in Veedhalai. These findings are clearly indicated in the Phylogenetic tree reconstruction based on direct sequences of nssrDNAusing MEGA-7 under Maximum likelihood criterion. The tree was statistically supported by bootstrap values (Figure 5 - see PDF).

The difficulties in analyzing endosymbionts using nssrDNA is challenging as reported by Toller et al. [41] Baker et al. [1] had reported that endosymbionts were classified based on nssrDNA, accordingly were classified as the endosymbionts A - I, the genus Durusdinium (Clade D) symbiont is most widely distributed in tropical waters [42-43] as well as detected in deep water corals, which may also occur in intertidal zone and coastal coral reef areas with environmental stress [44 - check with author]. The present study results were widely synchronized with symbiont diversity of Kenyan corals [45 - check with author]. The genus Durusdinium (Clade D) is a dominant endosymbiont in inshore region and its prevalence is increased by bleaching episodes which is more bleach resistant than the genus Cladocopium (Clade C)[46].

## Conclusion:

The corals have acquired the symbiont genus Durusdinium in their tissues and are more resistant under stress parameters such as temperature, salinity, and turbidity. Thus, the sequencing and the phylogenetic tree reconstruction of ss rDNA studies show the prevalence of the genus Durusdinium (Clade D) symbiont relationship with both fast growing (Acropora) and slow-growing (Favia, Favites and Porites) corals of Palk Bay, India. Thus, data shows the significant association of thermotolerant symbiont in the corals of Palk Bay, India leading to its natural conservation process of the coral community.
